# Cellular and Molecular Events in the Airway Epithelium Defining the Interaction Between House Dust Mite Group 1 Allergens and Innate Defences

**DOI:** 10.3390/ijms19113549

**Published:** 2018-11-10

**Authors:** Jihui Zhang, Jie Chen, Clive Robinson

**Affiliations:** 1 Institute for Infection & Immunity, St George’s, University of London, Cranmer Terrace, London SW17 0RE, United Kingdom; zhang.jihui@im.ac.cn (J.Z.); jchen99556688@outlook.com (J.C.); 2 State Key Laboratory of Microbial Resources, Institute of Microbiology, Chinese Academy of Sciences, Beijing 100101, China

**Keywords:** allergen delivery inhibitor, Der p 1, airway epithelium, asthma, TLR4, thrombin, alarmins, ADAM 10, tight junctions

## Abstract

Serodominant group 1 allergens of house dust mites (HDMs) are cysteine protease digestive enzymes. By increasing the detection of any allergen by dendritic antigen presenting cells, upregulating inflammatory signalling molecules, and activating cells crucial to the transition from innate to acquired immune responses, the proteolytic activity of these HDM allergens also underlies their behaviour as inhalant allergens. The significance of this property is underlined by the attenuation of allergic responses to HDMs by novel inhibitors in experimental models. The group 1 HDM allergens act as prothrombinases, enabling them to operate the canonical stimulation of protease activated receptors 1 and 4. This leads to the ligation of Toll-like receptor 4, which is an indispensable component in HDM allergy development, and reactive oxidant-regulated gene expression. Intermediate steps involve epidermal growth factor receptor ligation, activation of a disintegrin and metalloproteases, and the opening of pannexons. Elements of this transduction pathway are shared with downstream signalling from biosensors which bind viral RNA, suggesting a mechanistic linkage between allergens and respiratory viruses in disease exacerbations. This review describes recent progress in the characterisation of an arterial route which links innate responses to inhaled allergens to events underpinning the progression of allergy to unrelated allergens.

## 1. Introduction

Understanding the nature of allergens is an obvious facet of the discipline of allergy, yet detailed work on the enigma of allergenicity was relatively neglected until the last thirty years. Historically, one important reason for little engagement with the problem was the perceived technical difficulty posed by the challenge, and there can be no doubt that advances in molecular biology and the advent of the “omics” revolutions have provided previously unavailable capabilities which have transformed the understanding of allergenicity. Paralleling these developments, there has been a general renaissance in the field of innate immunity with a string of important discoveries revealing fundamental mechanisms which underlie the seamless escalation of low-level immunity into acquired responses with an allergic bias.

The initially slow pace of allergen characterisation is reflected by the fact that until 1984 there was no overarching approach to their denomination. That function now lies with a joint sub-committee of the International Union of Immunological Societies and World Health Organisation (http://www.allergen.org/). However, since then, there has been a significant acceleration in the systematic classification and comprehension of allergenic proteins largely driven by the enabling power of new research technologies. In fact, progress has been so dramatic that it is easy to form the impression that allergenic proteins are numerically large and highly diverse features of the protein universe. Remarkably this does not appear to be the case, and scrutiny of the AllFam database (http://www.meduniwien.ac.at/allfam/) actually reveals that <2% of protein families account for all known allergens, with most being limited to hydrolytic and non-hydrolytic enzymes, structural proteins, ligand-binding proteins, and miscellaneous enzyme inhibitors. This discrete clustering of allergens is intriguing from the dual perspectives of understanding fundamental mechanisms and the direction of this knowledge to health benefit. It becomes even more significant in the surrounding context of a phenomenon which operates within this clustering of allergen families which we have referred to as “molecular hegemony”. This phenomenon has been tacitly recognised for many years at a descriptive level in terms of the serodominance of a limited range of allergens from key triggers of disease, and as a functional principle it is supported by the concept of initiator allergens and the sequential events which occur in allergen polysensitisation [1,2]. Mechanistically, these phenomena can be explained by insights gained into the intrinsic bioactivities of allergens and the operation of processes which may be classed generally as adjuvancy, or more specifically as collateral priming, and which are illustrated by recent discoveries concerning the roles of type 2 innate lymphoid cells (ILC2s) and the innate activation of CD4^+^ T cells. [3,4,5,6]. That many, if not all, of the key events in these processes depend on allergens exploiting their normal functional bioactivity in their interactions with the human immune system provides a new understanding of why some substances are particularly potent allergens. This view has been referred to as the “functionalist” view of allergenicity [3]. The immunological principles which underlie this view are derived from predictive models and are becoming increasingly recognised as central elements of the origins and progression of allergic disease in humans.

This review will focus on group 1 allergens from house dust mites (HDMs) such as *Dermatophagoides pteronyssinus*, *D. farinae*, and *Euroglyphus maynei*. The principal reason for this emphasis is that the proteolytic activity of these allergens provides a potent mechanism to activate a wide range of innate effector pathways which drive both the initiation and chronic progression of disease. This has led to their bioactivity being recognised as a potential therapeutic target and the design of clinically-developable inhibitors of their effects [7]. Because group 1 HDM allergens are recognised as “initiator allergens” which can facilitate polysensitisation and its effects, their inhibition is likely to exert a previously unanticipated broad spectrum of benefits. It is also clear from work conducted on other clinically significant allergen sources where proteases form a part of the allergen repertoire (e.g., fungi) that similar protease-dependent events operate as central underlying processes of disease. Indeed, the evidence of cross-over between mechanisms identified for group 1 HDM and fungal allergens encourages the view that specific consequences of protease activity contribute strongly to generic events that are necessary for allergy [7].

House dust mites are sources of a particularly broad repertoire of allergens which are strongly associated with a wide range of allergic conditions [3,7]. Human exposure to HDM allergens occurs within indoor environments and is extensive for those with sedentary lifestyles, with significant consequences for the development of allergic disease [8]. Despite the large number of different allergens of HDM origin, there is compelling evidence that those of group 1 are of particular significance in promoting and maintaining allergic sensitisation to HDM allergens generally, and to other allergens from unrelated sources [7]. Through the remarkable scale on which they can activate innate immune responses and innate memory they are the chief exemplars of the molecular hegemony amongst allergens [3,7].

House dust mites are small arthropods which thrive in warm and moist environments, living on a diet of exfoliated skin flakes and other bio-debris which accumulates in house dust. Key to understanding the significance of the group 1 allergens was the sequencing of Der p 1 (the group 1 allergen from *D. pteronyssinus* and archetype for related allergens from other HDM species) and the bioinformatic indication that it might be a cysteine protease, a prediction subsequently verified experimentally [7]. That these HDM allergens are catalytically competent cysteine proteases is a function of their being gut-associated digestive enzymes which are then excreted as constituents of faecal pellets. This protease activity is pertinent to their role as allergy initiators because much human exposure to HDM allergens occurs through inhalation of HDM faecal pellets (these are of a respirable aerodynamic diameter) and from which the digestive enzymes are released rapidly in a highly concentrated form. Establishing the catalytic competence of these proteases was one of the first steps in developing the functionalist view of allergenicity [3]. In turn, it became the creation of a novel opportunity for an unprecedented approach to the treatment of allergic disease and a new class of small-molecule drugs which have been named allergen delivery inhibitors (ADIs) [7,9,10,11].

## 2. Group 1 HDM Allergens: Molecular Characteristics

**Figure 1:**
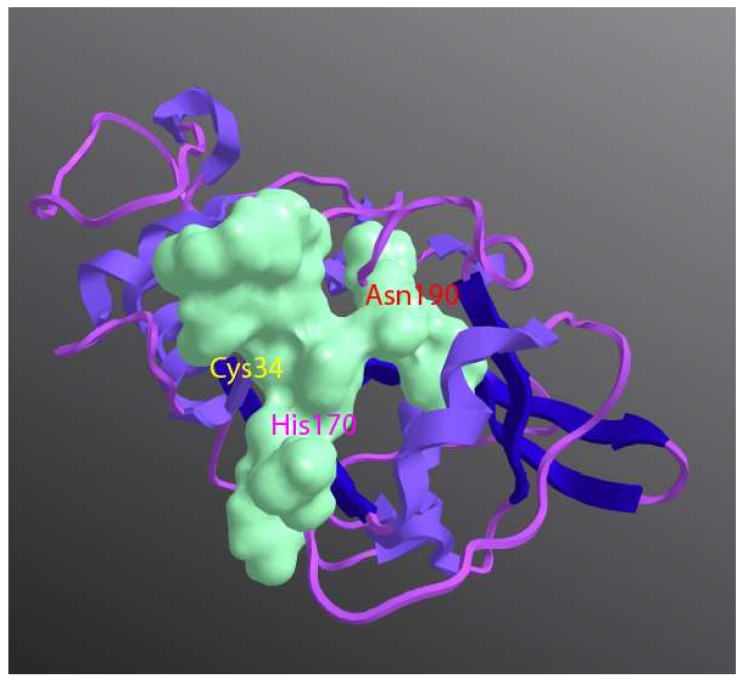
Structure of Der p 1 (Protein Data Bank (PDB): 2AS8; Der p 1.0105 isoform) with positions of catalytic residues shown. For clarity, a surface has been rendered over part of the substrate binding groove.

**Figure 2:**
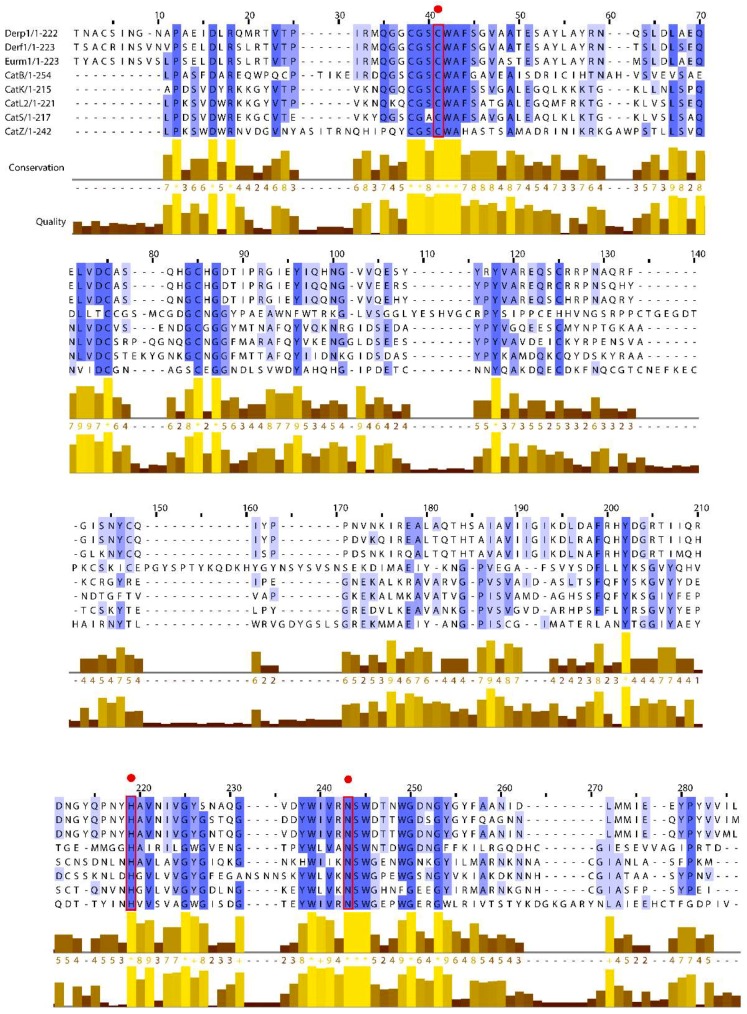
Multiple sequence alignments for the mature forms of HDM group 1 allergens from *Dermatophagoides* and *Euroglyphus* species with cathepsin family representatives (cat B, K, L2, S, and Z). Graphical representations of conservation and quality were created using Clustal Omega and Jalview. Threshold for conservation was set at 30%. Red rectangles and circles denote catalytic triad residues.

Group 1 HDM allergens are members of the C1 family of cysteine proteases belonging to the CA protease clan. Like the C1 family archetypes papain and cathepsins B, K, and S, their structures are characterised by two globular domains separated by a catalytic cleft containing the charge relay triad C^114^, H^250^, and N^270^ (Figure 1), but despite observable sequence-level similarities in the mature proteins (Table 1 and Figure 2) group 1 HDM allergens are properly to be regarded as a sub-family distinct from cathepsins because of structural and functional divergences in their prodomains [12]. In cathepsins which possess relatively long prodomains (e.g., cathepsin K, L, S), the propieces are formed from 2–3 α-helices containing a characteristic “ERFNIN” motif (EX_3_RX_2_ (I/V)FX_2_NX_3_IX_3_N), whereas this is lacking in cathepsin B whose prodomain is significantly shorter. After processing to the mature form of the protease, detached propieces with ERFNIN motifs retain an ability to inhibit their cognate enzymes. In contrast, the propiece of Der p 1 comprises 4 α-helices and differs from that of cathepsin B and those which contain a regular “ERFNIN” motif. Cleavage of proDer p 1, and by implication the corresponding zymogens from other HDM species, results in efficient proteolysis of the liberated propiece, thereby making it incapable of inhibiting Der p 1 catalytic activity in the mature 25 kDa protein [12]. The analogous group 1 allergen Blo t 1 from the tropical mite *Blomia tropicalis* appears to be an intermediate case between its *Dermatophagoides* counterparts and cathepsins; its prodomain contains the sequence “ERFQVN” as a modified “ERFNIN” motif and is also differentiated from the *Dermatophagoides* or *Euroglyphus* allergens in being a larger protein (~39 kDa vs ~25 kDa) with less obvious sequence identity or similarity overall. The modified ERFNIN motif prompts speculation that if Blo t 1 is catalytically competent (to date, this is inferred from a putative catalytic triad formed by C^119^ H^263^ N^283^ in homology modelling) its propiece may behave in a cathepsin-like manner and retain inhibitory capabilities when detached, perhaps providing some insight into why Blo t 1 is not serodominant, unlike its *Dermatophagoides* counterparts [7]. From the perspective of molecular allergology, the influence of the propiece in Der p 1 may extend beyond simply maintaining enzymatic latency in the immature form of the protein. The zymogen proDer p 1 is significantly less immunogenic than mature Der p 1, and this has been attributed to steric hindrance of important allergenic epitopes by the propiece [13,14]. As described later, the absence of proteolytic activity in the immature protein deprives the immune system of important adjuvant signals, and this further contributes to the comparatively effete nature of proDer p 1.

High-resolution structural data now exist for Der p 1 and Der f 1 which provide insight into the many similarities, and a few differences, between these group 1 HDM allergens [7]. Unsurprisingly, linear and/or conformational surface epitopes are conserved, consistent with non-discrimination between Der p 1, Der f 1, and Eur m 1 by a monoclonal antibody [15]. Significantly, immunoglobulin E (IgE) binding of group 1 HDM allergens may occur without penalty for proteolytic activity, potentially enabling IgE-bound allergens on mast cells and basophils to exercise parallel bioactivities, a property which is of interest given the ability of these allergens to activate mast cells in an IgE-independent, pseudo-allergic manner [16]. Among the differences are the propensity of Der p 1 to oligomerise in solution and some evidence of divalent cation binding. If these behaviours occur physiologically they may be relevant to biophysical interactions with mucosal surfaces, specifically their permeability properties in paracellular pathways (see Section 3.1). However, none of the reported differences have material bearing on enzymatic activity—a significant finding which underscores the conclusion from sequence and structure appraisals that they are a single target from the perspective of designing protease inhibitors [7]. The availability of crystal structures for these allergens supersedes a reliance on homology modelling, which was used in the early stages of protease inhibitor design for these targets and provides a structural refutation of claims that Der p 1 is a hybrid protease with both cysteine and serine protease activity [17,18]. Supporting the predictions of structural biology, the design of selective Der p 1 inhibitors has provided a powerful functional rebuttal which should finally draw a line under the hybrid protease concept which has confused some literature over an extended period. However, this episode illuminates one of many traps for the unwary in studies of allergen biology. The original suggestion of hybrid protease activity appeared in a paper in which low specificity tools were combined with an allergen preparation which was most probably of insufficient purity for the intended work [18].

Immunoaffinity chromatography has been used by several laboratories for the purification of Der p 1. One of the commonest trace contaminants of Der p 1 when purified by this technique is serine protease activity arising inter alia from allergens belonging to groups 3, 6, and 9, and rigorous steps are necessary to eradicate it. Partly for that reason, we have preferred a purification strategy for native Der p 1 which bypasses the requirement for enrichment by immunoaffinity chromatography and which incorporates multiple polishing steps for contaminant removal [9,19]. The ability to produce pure allergens to a rigorous and effective protocol is essential when they are being used as drug design targets, or when mechanistic analysis of biochemical or immunological signalling pathways is required. The possible presence of contaminants is a factor which is particularly important when using materials with undisclosed methods of preparation. For example, we have reported contaminant activity in a commercially-obtained sample of Der p 2, and although the contamination levels appear low they may nevertheless have the potential to lure the unwary to misleading conclusions [9]. The problem is not confined to allergens of HDM origin; we have also reported on particularly intransigent serine peptidase contamination of the serodominant cat allergen, Fel d 1 [20]. These difficulties further compound the problems in discovery immunology created by the use of commercial allergen extracts in models of disease processes. While the use of allergens in these mixture formulations has a greater real-world relevance to understanding their overall effects in vivo, there are issues which have the potential to impede the understanding of results and cause poor reproducibility of findings. Principal among these are the standardisation of source material and the use of manufacturing methods which are not specifically designed to preserve the sometimes fragile bioactive properties which are essential to allergenicity.

Growing appreciation of how the protease activity of group 1 HDM allergens interacts with innate immune responses, biochemical and biophysical defences provides a compelling case for targeting this activity with selective inhibitors [7]. The target validation of these proteases has been the subject of recent detailed review, and initial disclosures of developable inhibitors have been made [7,9,10]. These new molecular entities form the spine of an unprecedented class of agents known as allergen delivery inhibitors (ADIs) and inspection of their pharmacological profiles suggests a mixture of benefits, some of which were anticipated (e.g., inhibition of eosinophil, neutrophil and dendritic cell recruitment) and some which were not (e.g., inhibition of allergic bronchoconstriction) [9]. The new drugs are differentiated from steroids through mechanism of action and the range of pathways they influence, a combination which significantly attenuates the fundamental innate mechanisms which underlie the progression of disease [7]. An attraction of the group 1 HDM allergens which makes them a fascinating homologous target is that as proteases they activate high gain amplification signalling—in theory a single protease molecule could eventually attack all cleavable targets on a cell—and thus its prevention should bring significant benefits [7,10,11]. The homology between the proteases makes it feasible for an inhibitor designed against the group archetype to be an effective inhibitor of group 1 allergens from other HDM species, which is a further advantage [7,10].

## 3. Group 1 HDM Inhalant Allergens and Intercellular Adhesion in the Airway Epithelium

Inhalant allergens from HDM begin their interaction with immunological sentinels when they impact on the airway mucosa and contact the airway lining and cells resident in the airway lumen. In that sense, a principal component of allergic asthma may be considered to originate in the airway epithelium because events at this interface are influential in shaping the subsequent development of disease, and the recapitulation of these events is a central feature of the chronic effects which define attendant hallmark pathophysiology.

The sophisticated defence system of the airway lining can detect potential pathogens and mount cellular and humoral responses against them when appropriate. This capability comprises passive, or more correctly structural, and reactive components. The operative structural component is the airway lining itself because the epithelial cells from which it is formed dictate the flow of molecules and cells across it. Only those materials that are cell permeant, or which benefit from the availability of transporter systems, can cross the airway epithelium by a transcellular routing; other materials must either rely on paracellular movement or contact other cells which themselves use the paracellular space to enter and leave the airway lumen. For materials which may be endocytosed, the extent of transepithelial transfer of the intact molecule may be small due to endosomal processing within cells, so increased paracellular permeation provides an opportunity for their bulk transepithelial delivery in an intact form. Understanding how allergens interact with the airway epithelium and the routes by which they cross this barrier is, therefore, important in defining the exact extent to which mucosal surfaces, such as the airway epithelium, shape the development of allergy.

Dendritic cells (DCs) are the primary antigen presenting cells in the lung [21,22,23]. While some quiescent DCs possess dendrites which infiltrate the airway epithelium, the majority have a sub-epithelial localisation but increasingly penetrate the airway lumen in disease [24,25]. Similar observations have been made concerning the behaviour of Langerhans cells in skin [26]. Consequently, the opportunity for DC-driven humoral responses potentially increases as a function of epithelial permeability. Allergen uptake into epithelial cells could form an alternative route to antigen presentation, but secondary antigen presentation pathways such as this result either in the development of tolerance, or are simply less significant compared to antigens that can present via DCs [27,28,29,30].

### 3.1. Tight Junctions and Group 1 HDM Allergens

Paracellular permeation of ionic and non-ionic solutes across epithelial surfaces is regulated by interepithelial tight junctions (TJs) which are formed from contiguous rings of cell adhesions at the apical pole of epithelial cells. Tight junctions are supramolecular assemblies of proteins which occlude entry to the paracellular space between adjacent epithelial cells. They comprise a combination of membrane-spanning adhesion molecules (MARVEL domain proteins (occludin, tricellulin, and MARVELD3), the claudin family proteins, and junction-associated molecules (JAMs)) together with intracellular proteins of the TJ plaque (e.g., zonula occludens (ZO)-1, -2, -3) which provide connections to the cytoskeleton [31]. Regulated ion flow through TJs occurs via permselective gated pores in the junctional complex, whereas the diffusion of uncharged solutes utilises other mechanisms which accommodate molecules up to a Stokes’ radius of ca 2 nm. This size limit, if correct, could be enough to allow a restricted transepithelial flow of low molecular weight allergens, but experimental data show that little transepithelial flux of Der p 2 (~14 kDa) occurs in the absence of the proteolytic activity of Der p 1. This suggests that the transepithelial disposition of these allergens requires more than a compliant molecular radius, and that an ensemble of other factors (oligomerisation, binding, shape, polar surface area, protein hydration shells) may dominate functional behaviour [9]. In fact, the nature of this size-selective pathway may simply enable protease allergens such as Der p 1 to gain a more rapid access to the adhesion proteins for the initiation of TJ cleavage.

Claudin family proteins share a basic topological similarity with MARVEL family proteins, such as occludin, in having intracellular N- and C-termini, and possessing two extracellular domains (for review see: [32]). These extracellular domains show notable similarity in “classical” claudins, indicative of important conserved functions, while their differences are likely to encode molecule-specific functional attributes. In the case of occludin, the second extracellular domain is compositionally diverse, whereas the first domain is rich in Gly and Tyr residues, suggesting that the two domains serve different functions. The intracellular C-termini of both families interact with ZO-1, ZO-2, and ZO-3, which are members of the membrane-associated guanylate kinase (GUK)-like proteins comprising part of the intracellular TJ plaque [31]. Occludin interacts with the GUK domain of these proteins, while claudins and JAMs interact with their PSD95/DL9/ZO-1 domains [32,33,34,35].

The extensive repertoire of claudin family proteins and their manner of interaction potentially enables their creation of junctional adhesions with highly nuanced functional properties. Individual claudins are known to form cis-interacting polymers within the same cell and polytypic trans-interactions between adjacent cells, with heteromeric and heterotypic interactions adding further levels of complexity (although certain hetero-interactions are notably disfavoured). The complexities of these interactions, which are still not well understood, are relevant to the categorisation of claudins according to the properties of their particular sub-family members (viz: sealing-, cation-selective or anion-selective types), but the overall functional behaviour of TJs at tissue level may be shaped and modified by claudins from different sub-families also present in those junctions. This complexity may be of some significance in tissue repair, where the ability to undertake compensatory remodelling of junctions could be of value in defence or transitional phenotypic change in the epithelium. These aspects are relevant to the consequences of TJ cleavage evoked by protease allergens.

The regulation of non-ionic paracellular permeability is an important function of occludin [36,37]. Occludin is a target substrate of Der p 1 in airway epithelial cells and its proteolysis results in TJ cleavage which is accompanied by a non-specific increase in epithelial permeability [38,39]. Selective inhibition of Der p 1 by novel, high potency inhibitors prevents the increase in epithelial permeability and attenuates the ability of mixed HDM allergens to evoke the production of IgE to HDM allergens [9,40]. Consistent with this, Der p 2, which is also classed as being serodominant, relies on the proteolytic activity of Der p 1 for its transepithelial passage. Putative cleavage sites for Der p 1 have been identified in both extracellular domains of occludin and proteolysis is further facilitated by an intracellular mechanism which initiates cleavage proximal to the C-terminus [39]. These effects are rapidly reversible and the junctions potentially reinstatable over a period of hours because de novo synthesis of occludin occurs quickly after the initiation of TJ cleavage [39]. The reversibility of these events underlines an important, and sometimes misunderstood, point about these interactions between allergens and epithelial cells. Tight junctions are not static, unchanging entities, but dynamic assemblies with an equilibrium state in which junctional integrity is maintained against a steady background of continuous protein replacement or remodelling [41,42]. The effect of Der p 1, or other protease allergens, is to temporarily shift this equilibrium such that the rate of protein degradation exceeds its replacement. However, on termination of the inciting stimulus the epithelium returns to its original balance of TJ protein synthesis and degradation, resulting in the restitution of TJ integrity [39]. In terms of mechanism and scale, this is a fundamentally different process from the epithelial damage elicited by chronic inflammation due to inflammatory leukocytes, and consequently, requires suitably sensitive techniques to appreciate the subtleties involved. In the landmark investigation of this problem the tandem use of 2-photon molecular excitation microscopy (then in its infancy as a tool for biologists) coupled with quantitative kinetic analysis of junctional components in 3-dimensional isosurface reconstructions of cells, was crucial in revealing these events [38,39]. Before leaving the topic of junction re-assembly following HDM allergen exposure it is worth noting that, while not to our knowledge previously subject to investigation, JAMs may too have a role in this process given that their interaction with intracellular TJ proteins (e.g., ZO-1, partitioning defective) and associated signalling proteins such as atypical protein kinase C, Rho-associated coiled-coil kinases, and Rho GTPases.

Unlike occludin, with which they associate in TJs, claudins are considered to have a significant role in regulating the ionic permselectivity of epithelia and are thus key to the development of transepithelial electrical resistance (TER). Although a commonly-used surrogate for solute permeability, TER arises from the presence of ion-selective pore-forming claudins in TJs, and thus it is feasible to observe significant dissociation between TER and solute permeability. A striking demonstration of this is the low resistance variant of the Madin–Darby canine kidney cell line which fails to develop a significant TER, despite having morphologically identifiable TJs and discriminating the permeability of macromolecular solutes in the manner expected for cells bearing TJs. The suitability of TER as a permeability surrogate has relevance when studying the interaction of allergens with epithelial cells, particularly under conditions where TJ composition may be remodelled because of pathophysiological events.

Claudins are responsible for the characteristic appearance of strands seen in TJs when imaged by freeze-fracture and are thus distinguishable from occludin which does not form equivalent structures. The number of claudin family members suggests that their sequence diversity encodes specific functions and/or results in their localisation in specific tissues—claudin-18 has particular expression in the lung, for example [43,44,45,46]. Claudins are themselves putative targets of protease allergens from HDM, with cleavage sites proposed for the serine protease allergens in both extracellular domains of claudin-1 [47]. Elsewhere, we have reported a more detailed examination of the cleavage of the first extracellular domain of claudin-1 by the cysteine protease Der p 1 [9]. Claudin-1 was used in those studies as a family archetype of claudins expressed by human airway epithelial cells (i.e., claudins -1, -3, -4, -5, -7, and -18), exploiting the fact that the extracellular domains of claudins are their regions of greatest similarity [46,48,49,50]. In being representative of the “sealing” type of claudins, which are functionally distinguishable from their anion- (e.g., claudin-17) and cation-selective (e.g., claudin-2, -10, and -15) relatives, claudin-1 and its counterparts are likely to be of greater relevance to the allergen delivery effects of protease allergens. This focus on “sealing” type claudins is justifiable in the present context because airways express relatively low levels of pore-forming claudins [45]. Our studies with Der p 1 and claudin-1 have revealed that (i) some of these cleavage sites are susceptible to both cysteine and serine peptidase HDM allergens, (ii) the cleavage sites appear to be conserved across a wide range of claudins, and (iii) a notable cleavage site in claudins that is susceptible to both serine and cysteine protease HDM allergens is also present in occludin, suggesting that the Leu-Leu dyad might be a particular “hotspot” for interaction with protease allergens [9,39,47]. This raises the question of whether these dyads constitute a target for other pathogens because pathogen-induced junctional deficits are now recognised to underlie several diseases [51,52,53,54]. From the perspective of the production of reactive oxidant species (ROS) in the airway epithelium by group 1 HDM allergens, a topic we shall visit later, it is interesting to note that deficiency in the antioxidant regulator nuclear factor (erythroid-derived 2)-like 2 (Nrf2) leads to enhanced responses to allergens, whereas its over-expression protects against them and enhances the expression of ZO-1, occludin, and E-cadherin [55,56,57,58,59,60,61].

Based on its detection in the gut, it has been proposed that Der p 1 might contribute to the pathogenesis of chronic inflammatory diseases in the absence of an allergic response [62]. Evidence suggests that an important component of this non-allergic mechanism involves the cleavage of TJs in gut epithelium, in a manner akin to that shown in airway epithelium, occurring together with the release of inflammatory cytokines (tumour necrosis factor α (TNFα) and interleukin-10 (IL-10), and breakdown of protective mucus by the enzymatic action of Der p 1 [62]. If correct, these findings raise the question of whether an oral route for sensitisation to HDM allergens might exist, especially in early life at a time when innate immune responses play decisive roles in shaping the development of allergy. 

To the best of our knowledge, other TJ adhesion proteins have not been studied for their susceptibility to protease allergens. Tricellulin and MARVELD3 are, like occludin, members of the TJ-associated MARVEL domain protein family (TAMPs) which have an affinity for membrane microdomains rich in cholesterol. Of these other TAMPs, tricellulin is concentrated in TJ rings where three cells abut, and it is speculated that its function is to stabilise interepithelial junctions at points which are under higher structural stress than most contacts, which are bicellular. However, as we show elsewhere [9], the first extracellular domains of tricellulin and MARVELD3 lack known cleavage motifs for Der p 1, suggesting that they might be resistant to extracellular attack. This does not, however, exclude their vulnerability to intracellular processing like that which occurs in the C-terminal domain of occludin in response to Der p 1 [39].

Cleavage of TJs by HDM protease allergens is just one example of how they influence epithelial permeability because indirect effects have also been identified. Claudin expression generally is downregulated by IL-13, and specifically it results in the reduction of claudin-18 whose tissue localisation is strongly associated with the airways [46]. The cytokine IL-13 is, in turn, upregulated by Der p 1 in a protease-dependent manner and this can be prevented by allergen delivery inhibitor compounds. Other cytokines, notably TNFα, also have downregulatory effects on TJ adhesion proteins [63,64]. Naturally, the consequences of IgE-dependent mast cell activation and inflammatory cell recruitment enable these and additional mechanisms for epithelial barrier impairment to be brought to bear. Furthermore, the response to viral infection of the airways provides other routes to epithelial dysregulation which are relevant to the exacerbation of disease [53]. The role of ROS in linking the proteolytic effects of Der p 1, the activation of biosensors for viral RNA, and the upregulation of inflammatory responses will be discussed later, but it is worth noting en passant that ROS generation has implications for the epithelial barrier because protection against ROS, by over-expression of the antioxidant regulator nuclear factor (erythroid-derived 2)-like 2 (Nrf2), enhances the expression of occludin, ZO-1 and E-cadherin [55].

A similar paradigm operates in skin which, unlike the surface of the lung and nose, is a complex stratified epithelium. House dust mite protease allergens such as Der p 1 increase the permeability of the epidermis and delay its repair by releasing inflammatory cytokines [65,66,67,68,69]. An inverse relationship exists between the levels of claudin-1 and Th2-polarised responses in atopic dermatitis, with the effects on TJ adhesion protein expression extending to non-lesional skin [70,71]. Functionally, an immunological consequence of TJ cleavage in keratinocytes is to enable Langerhans cells (the major antigen presenting cells in skin) to forge TJ adhesion molecule-dependent intercellular contacts which allow increased antigen sampling while maintaining overall integrity of the barrier [26].

Whereas the engagement of Der p 1 with TJs has been revealed at the scale of potential cleavage sites in adhesion proteins, and by showing the influence of Der p 1-inducible cytokines on junctional integrity, there remains only a poor understanding of the detailed mechanisms which link TJ cleavage with increased permeability to macromolecules and cells. In large part this reflects the lack of a good mechanistic model of the macromolecular permeability barrier function of epithelia, a position which sharply contrasts with the better understanding that exists for TJ gating of ionic permeability. Given the strategic importance of these processes, further effort should be applied to gaining a better understanding of the general permeability function of TJs, and, more specifically, explore these events as a checkpoint in allergy.

Examination of the TJ plaque reveals its connections with a variety of signalling and response mechanisms, notably actomyosin. Therefore, TJs offer a potential for mediating both inside-out and outside-in cell signalling, and these processes may be important in regulating TJ function. However, some epithelial cell responses which superficially associate with TJ cleavage are not transduced through them. An example is the induction of apoptosis by Der p 1 which requires the catalytic competence of the allergen, but which can occur in cell lines constitutively lacking TJs, or in cells where they have been removed by a calcium switch procedure [72]. As discussed later, both the group 1 HDM allergens and ligands of viral RNA biosensors trigger the production of ROS in airway epithelial cells in a process which involves the activation of myosin motors. However, it is currently unknown whether this activation affects TJ integrity and contributes to TJ cleavage initiated by direct proteolysis of occludin and claudins.

Adapter proteins, the most studied of which is ZO-1, are compositionally important elements of TJ plaques responsible for transducing the signalling function of TJs. Disruption of ZO-1 occurs following exposure of airway epithelial cells to Der p 1 [39], implying that the signalling function of TJs is affected as a consequence. As described earlier, ZO-1 interacts with claudins, occludin, and JAMs, but these interactions are supplemented by others subserving roles in cell signalling and gene expression. An example is ZO-1-associated nucleic acid binding protein (ZONAB) which regulates gene transcription and cell proliferation [73]. A striking feature of the adapter proteins associated with TJs is the association between particular adhesion proteins, adapter proteins, and signalling pathways associated with TJs, implying strong demarcation of function. While many of these are involved with epithelial “maintenance” (e.g., cell proliferation, junction assembly, and determination of cell polarity) [74], a number relate to pathways relevant to inflammatory responses. For example, occludin is involved in signalling via mitogen activated protein kinase (MAPK) pathways [75], and the related TJ protein, MARVELD3, has a signalling function involving mitogen activated protein kinase kinase kinase 1 (MEKK1) which appears to dynamically regulate cell proliferation and is thus relevant to tissue repair [76]. The extent to which these pleiotropic signalling pathways might provide a coupling with mediator release is an interesting, but still relatively unexplored question. However, whether signalling into inflammatory events via TJs is a dominant or even necessary factor seems unlikely because epithelial cell lines devoid of TJs generate a wide range of mediator outputs upon suitable stimulation by group 1 HDM allergens, although this does not exclude signalling from TJs exerting a significant modulatory role on such events. 

### 3.2. Adherens Junctions and Group 1 HDM Allergens

Adherens junctions (zonulae adherentes, ZAs) are expressed by epithelial cells in close association with TJs. These ZAs form homotypic adhesions with neighbouring cells, but unlike TJs, they do not occlude the paracellular channel. The key transmembrane component of ZAs in the airway epithelium is E-cadherin, the extracellular portion of which comprises five repeating homologous domains while the intracellular domain has binding sites for catenins to provide a linkage with the actin cytoskeleton. E-cadherin also exists outside of ZAs where its role extends beyond canonical homotypic adhesion. Inspection of the extracellular domains of E-cadherin suggests multiple sites of vulnerability to HDM protease allergens, but experimentally the proteolysis of E-cadherin by HDM protease allergens in epithelial cells proceeds more slowly than that of the TJ adhesion proteins. This behaviour is understandable from steric considerations where, until TJs are derogated by proteolysis, their presence impedes access to E-cadherin in ZAs. However, E-cadherin may be targeted by other proteolytic mechanisms that are activated consequentially by Der p 1 and which are less subject to that constraint. Among these other mechanisms is the activation of a disintegrin and metalloprotease (ADAM) 10, well-recognised as a major “sheddase” of E-cadherin and a signalling component in the generation of ROS by HDM allergens, specifically Der p 1 [77,78,79].

### 3.3. Desmosomes and Group 1 HDM Allergens

Not all interepithelial junctions are primary cleavage targets of HDM protease allergens. Desmosomal adhesions, which provide epithelia with resilience to physical stress rather than controlling epithelial permeability, are examples of this. Desmosomes comprise the single membrane pass desmocollins and desmogleins which are anchored to plakoglobin and desmoplakin in the desmosomal plaque and thus bridged to keratin intermediate filaments. Unlike TJs and ZAs, desmosomes do not appear to be cleaved by Der p 1, but instead show increased staining after allergen exposure [39]. The molecular mechanism of this effect and its significance remain unknown.

### 3.4. Consequences of Intercellular Junction Cleavage by HDM Allergens

Disruption of the major junctional adhesions of epithelial cells has consequences which reach beyond “simple” effects on permeability to allergens. While not all the available literature provides examples at the airway interface, the principle is established at other mucosal surfaces and reasonable grounds exist for its replication in the lung. Networks of dendritic antigen presenting cells, which have close affiliations with the epithelial barrier are positioned to react to these changes, as are infiltrating cells attracted by chemokines. Dendritic cells and γδ intraepithelial lymphocytes express occludin and/or claudin proteins which are linked to antigen sampling and migration to lymph nodes [26,80,81,82,83]. Furthermore, DCs are reciprocally regulated by epithelial cells in an E-cadherin-dependent manner, indicating that disruption of E-cadherin binding should lead to cellular activation [84], a view encouraged by the observation that production of thymic stromal lymphopoietin (TSLP) is inversely related to E-cadherin expression [85]. As described above, E-cadherin proteolysis can be achieved directly by Der p 1 when the steric hindrance by TJs is reduced by their cleavage, but an endogenous mechanism for E-cadherin proteolysis is known and operates through ADAM 10, which may underpin the principal mechanism for E-cadherin shedding [77]. Our studies of human airway epithelial cells have identified ADAM 10 activation as a key step in a cyclical signalling pathway activated by the proteolytic action of Der p 1 and by ligation of biosensors which detect viral RNA [78,79]. These observations, together with emerging understanding of ADAM 10 in allergy (see Section 4.4) place these exogenous and endogenous proteolytic events, and changes in E-cadherin binding properties, at the centre of an intriguing signalling nexus which links allergy development with its progression. Illustratively, E-cadherin expression is of relevance to crosstalk between epithelial cells and ILC2s which express killer cell lectin-like receptor sub-family G, member 1 (KLRG-1), a binding partner of E-cadherin. Ligation of KLRG-1 prevents the production of IL-5 and IL-13 by ILC2s, so disruption of this inhibitory state may be a required event for ILC2 activation [86] and preparation of the transition from innate to acquired immunity. Other actions of ADAM 10, such as the cleavage of CD23, IgE biosynthesis and the spatial positioning of mast cells in tissues, are also relevant to this transition (see Section 4.4).

## 4. Innate Effects of Group 1 HDM Allergens

In addition to effects on TJs, the proteolytic actions of group 1 HDM allergens have been implicated in many other actions pertinent to the initiation and maintenance of allergy. Broadly summarised, the majority are related to signal transduction mechanisms which polarise towards T_H_2 immunity. These include the generation of thrombin, release of ATP (and other purinergic or pyrimidinergic mediators), the formation of ROS and reactive nitrogen species (RNS), the upregulation of chemokine and cytokine expression and the release of other inflammatory mediators. For some pathways the initiating events are easily understood because they involve cleavage of a target protein which leads either to its activation or inactivation, e.g., CD23, CD25, α_1_-antitrypsin [87,88,89]. By analogy, the processing of IL-33 also fits into this category because Der p 1 and papain (which is, of course, an allergen in its own right as well as a surrogate of group 1 HDM cysteine protease allergens) execute gain of function cleavages between A^95^ and H^109^ to yield a truncated form of IL-33 (principally comprising its C-terminal IL-1 cytokine domain) [90]. Similar cleavages of full length IL-33 to super-active forms have been reported with fungal extracts containing protease allergens [90] and neutrophil- or mast cell-derived proteases [91,92]. Given the association of IL-33 with allergy, these observations suggest that the ability to release and process IL-33 may underpin the initiator role of protease allergens.

For other effects, such as the stimulation of IL-6 or IL-8 production, the activation mechanism has been less clear [93,94]. The discovery of the tethered ligand family of protease-activated receptors (PARs) which are primarily responsive to serine proteases—and therefore anticipated targets of Der p 3, Der p 6, and Der p 9—prompted interest in them being activated non-canonically by cysteine protease group 1 HDM allergens. Activation of PARs by peptide agonists which mimic the tethered ligands of these receptors results in cytokine production, giving such a mechanism plausibility, at least for the serine peptidase allergens [95]. Interest in PAR activation by HDM allergens was provided with further support on finding that one of the PAR family receptors, PAR2, was upregulated in asthmatic airways [96]. However, several observations now cast doubt over how important PAR2 cleavage might be for transducing responses to HDM allergens, especially those of group 1. Illustrative of a broader body of literature, evidence from animal models points to PAR2 exerting both cytoprotective and inflammatory effects according to circumstance [97,98]. Furthermore, while PAR2 has some involvement in responses to HDM allergens, it seems inessential to allergy development by the inhaled route because sensitisation and challenge responses are not ablated in PAR2-deficient mice [99]. As will be discussed later, these observations concerning Der p 1 and PAR2 should not be interpreted to indicate that PARs generally are unimportant in the airway response to Der p 1, or that PAR2 is without any role.

By means of elegant studies in mice, other investigations have revealed that epithelial responses are indispensable in the sensitisation to HDM allergens and that Toll-like receptor 4 (TLR4) is essential to this process. Given the similarity of the group 2 HDM allergens to MD2/lymphocyte antigen 96 which is involved in the activation of TLR4 by bacterial lipopolysaccharide (LPS), one explanation for the centrality of TLR4 to HDM sensitisation is that Der p 2 facilitates TLR4 activation by LPS [100]. Lipopolysaccharide is of course inextricably linked with HDM allergens as a component of house dust, making this is an interesting concept given the associations of LPS endotoxin with allergy. Other HDM allergens (e.g., groups 22 and 35) bear relationship to MD-2, and yet others (e.g., groups 5, 7, 13, 14, and 21) have ligand-binding potential for lipids generally, if not LPS per se, apparently reinforcing the hypothesis. However, without a means of connecting group 1 HDM allergens to TLR4 activation, this explanation for the indispensability of TLR4 conflicts with the efficacy of Der p 1 inhibition in preventing the development of allergic sensitisation [9,40,101,102].

A resolution of this paradox has been proposed following the unexpected discovery that Der p 1 (and by inference its other group 1 homologues) behaves as a prothrombinase which is capable of forming thrombin without upstream components of the coagulation system being activated [7,103]. Experimental inhibitors with high potency against Der p 1 (i.e., the allergen delivery inhibitor compounds ADZ 50,000, ADZ 51,529, and SGUL 1773) prevent this effect and its downstream consequences, confirming they are due to the cysteine peptidase action of Der p 1 [7,79,103]. The ability of Der p 1 and other group 1 HDM allergens to stimulate thrombin production in the airway epithelium provides a mechanism for these allergens to evoke the canonical activation of PARs. In human airway epithelial cells, this stimulation by Der p 1 involves both PAR1 and PAR4, which were previously considered incapable of direct activation by group 1 HDM allergens [94]. While the involvement of PAR1 and PAR4 is anticipated because both can be activated by the thrombin formed by Der p 1, the action of Der p 1 is interesting in being fully blocked by antagonists of either PAR1 or PAR4 (e.g., SCH 79797, FR 171113, tcY-NH_2_) and, consistent with this, gene silencing of either receptor markedly attenuates the ensuing response [103]. The surprising ability of antagonists of either receptor to fully inhibit the cellular response is compatible with a model for co-operative hetero-oligomerisation of PARs and the formation of a ternary complex between PAR1, PAR4, and thrombin [7]. The notable mechanistic gain from the formation of this ternary complex is enhanced receptor activation because the interaction of thrombin with PAR1, which contains a thrombin-binding exosite in addition to presenting a cleavage site in the extracellular N-terminus of the protein, facilitates presentation of thrombin to PAR4 which lacks a thrombin exosite [104]. The activation of thrombin formation by this innate response to Der p 1 provides a new explanation for the presence of elevated levels of thrombin in airway surface liquid in asthmatic airways [105,106,107,108,109,110], a phenomenon previously thought simply to represent the operation of tissue repair processes activated by IgE-dependent inflammation. After allergen exposure, thrombin activity in asthmatic airways can rise to levels capable of driving cell proliferation, implying a role in tissue remodelling [110]. That significant amounts of thrombin could be generated by an entirely innate response to a major environmental allergen and the response to respiratory RNA viruses [111], suggests that thrombin-dependent smooth muscle proliferation, which may have functional consequences for the responses to any inhalant allergen, can occur regardless of events mediated by IgE cross-linkage [103]. If correct, a corollary is that a component of allergen-dependent airway hyperreactivity may result from innate, IgE-independent pathophysiology which can be episodically amplified by IgE-mediated exacerbations, or reinforced by reactions to viral RNA [78]. Two puzzles which arise from this proposition are the extent to which the mechanism may be activated in people without allergic sensitisation and why people with asthma may be more susceptible to its effects.

PAR1 is upregulated by respiratory viral infections and contributes to the pathogenicity of influenza A [112,113], observations potentially relevant to exacerbations of asthma caused by interactions between allergens and RNA viruses such as rhinovirus, respiratory syncytial virus, and influenza. The ability to block one aspect of this interaction through inhibition of the protease activity of HDM allergens suggests a new approach to the treatment of asthma exacerbations, especially as achieving this by selective targeting of the virus response pathways lacks efficacy [114]. As will be discussed later, there are additional aspects of signal transduction activated by group 1 HDM allergens which provide signalling convergence with innate responses to RNA viruses. 

### 4.1. Der p 1, PARs, ROS, and Reactive Nitrogen Species (RNS)

The canonical activation of PAR1 and PAR4 by the prothrombinase activity of Der p 1 in airway epithelial cells is a precursor to the generation of ROS and RNS [78,79,103]. The formation of these reactive signalling intermediates provides a basis to explain two conundrums. Specifically, it offers a rationale behind the ability of Der p 1 to elicit IgE-independent cytokine production through mechanisms involving the activation of (i) redox-sensitive transcription factors, (ii) signalling cascades strongly associated with allergy (e.g., mitogen-activated protein kinases (MAPK) and the signal transducer and activation of transcription (STAT) family), and (iii) DNA damage [115,116,117]. More generally, it suggests that some individuals may be at greater risk of developing sensitisation to group 1 HDM inhalant allergens because of genetically-encoded deficits in antioxidant defences, or vulnerabilities to ROS/RNS which are triggered by the innate cellular responses to these allergens [116,118,119,120,121,122,123,124,125]. A link between oxidative stress and allergic sensitisation has been explored in experimental models which reveal the potential for significant interactions between certain allergens and oxidative responses that are associated with disease pathways. For example, deficiency of the antioxidant regulator nuclear factor (erythroid-derived 2)-like 2 (Nrf2), or an impaired ability to upregulate it, enhances responses to ovalbumin or HDM allergens [56,57,59,60]. Conversely, activation of this master regulator protects against IL-33 release and allergic responses [58]. Papain, like Der p 1 a C1 family protease and an occupational allergen in its own right, requires oxidative stress to initiate allergic sensitisation, and attenuating the protease activity of HDM allergen extracts (a means of preventing ROS formation through thrombin-dependent activation) inhibits allergic inflammatory responses [59,126].

It is likely that ROS generation induced by Der p 1 occurs in various subcellular locations and involves multiple members of the NADPH oxidase family of enzymes. However, some of the ROS production occurs at the level of electron transport in mitochondria through the two-electron-dependent reduction of oxygen to form superoxide anion [103]. Activation of airway epithelial cells by Der p 1 also induces nitric oxide synthase and the formation of nitric oxide. Nitric oxide reacts with superoxide anion to form peroxynitrite, a product which is readily reactive with the dihydrorhodamine probe used to study these responses indicating that previously-reported effects represent a composite measure of ROS/RNS responses [78,79,103].

### 4.2. Der p 1, PARs and Epidermal Growth Factor Receptor Signalling

**Figure 3:**
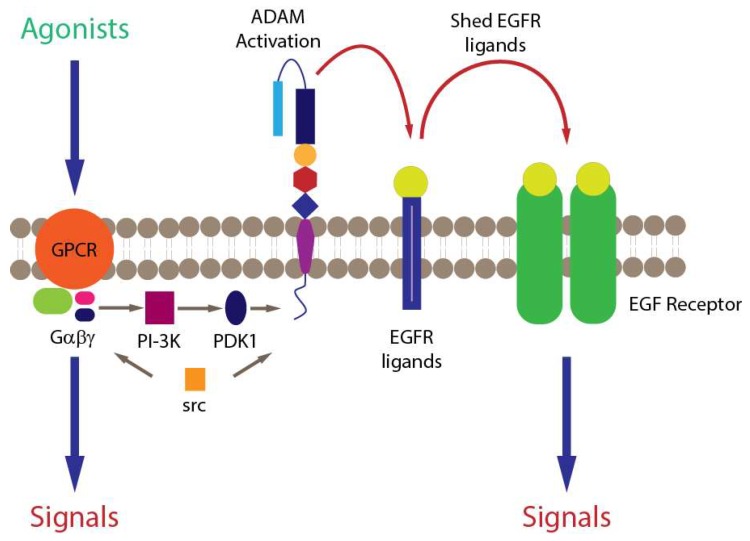
Mechanisms of signalling crosstalk between GPCRs and EGFR (Abbreviations: GPCR, G-protein coupled receptor; PI 3-kinase, phosphoinositide 3-kinase, PDK 1, phosphoinositide-dependent protein kinase 1; src, proto-oncogene tyrosine-protein kinase src; ADAM, a disintegrin and metalloprotease; EGF/EGFR, epidermal growth factor/receptor). Blue arrows denote initial agonist-receptor interaction and the signals derived directly or indirectly from it. Grey arrows denote intracellular signalling events involved in crosstalk. Red arrows denote extracellular signaling crosstalk.

**Figure 4:**
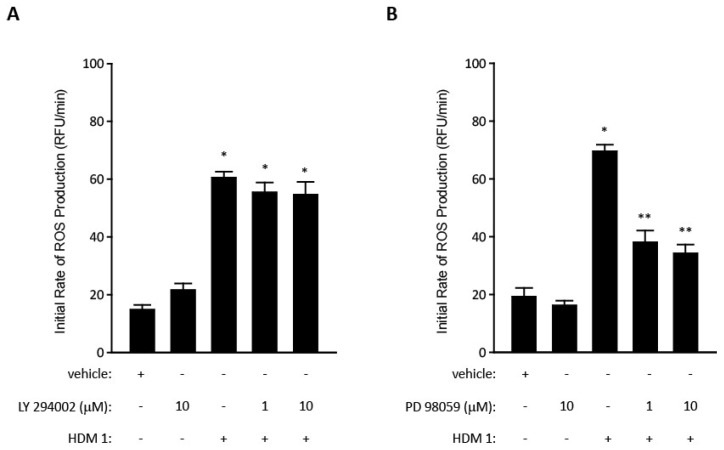
Effects of LY294002 (PI 3-kinase inhibitor) and PD98059 (mitogen activated protein kinase kinase inhibitor) on HDM-allergen stimulation of ROS generation in human airway epithelial cell line Calu-3. Data are shown as mean ± standard error values (*n* = 8). * *p* < 0.001 vs vehicle controls; ** *p* < 0.001 vs allergen-activated cells (1-way ANOVA with post-hoc testing by Student Newman Keuls procedure). The ROS production was quantified in cells loaded with dihydrorhodamine 123 as described in [78,103].

The ability of mixed HDM allergens, specifically their group 1 component, to stimulate ROS/RNS production through proteolytic activation of PARs raises questions about the signalling events which couple these important steps [7,103]. One feature of PARs, like many G-protein coupled receptors (GPCRs), is their transactivation of other receptors through signalling cross-talk. Examples of PAR cross-talk have been found with nucleotide-binding oligomerisation domain-like receptors, cargo receptors, some TLRs, other GPCRs, ion channels, and receptor tyrosine kinase (RTK) family members. Receptor tyrosine kinases are of interest in this communication spectrum because the family archetype is epidermal growth factor receptor (EGFR), which through pleiotropic roles in cellular function, is unsurprisingly incriminated in asthma. The archetypal mechanism of cross-talk between GPCRs and EGFR is well understood (Figure 3), which led us to investigate whether EGFR transduces signalling from PAR1/4 in the Der p 1-dependent production of ROS by epithelial cells. These studies showed that unlike the archetypal case, responses to Der p 1 lack a significant involvement of phosphoinositide-3-kinase (PI-3-kinase) because they are poorly sensitive to the inhibitor LY294002, whereas they are inhibited by the MAPK kinase inhibitor PD98059 (Figure 4). Nevertheless, there appear to be at least two instances of EGFR activation in the signalling cascade activated by Der p 1 in airway epithelial cells, one involving ADAM 17 and the other ADAM 10. The difference between them is that the ADAM 10-dependent component is activated by the release of ATP and the ligation of P_2_X_7_ and P_2_Y_2_ receptors, whereas the ADAM 17-mediated activation of EGFR operates upstream from ATP release [78]. The concentration of ATP in airway surface liquid is elevated in asthma through several mechanisms, supporting an association with disease, especially through the underlying activation of innate immune signalling [127]. As an initiator, director, and maintainer of allergic inflammation, ATP triggers the release of IL-33 and activates dendritic cells to programme allergic responses [128,129], while in established allergy it augments IgE-dependent mediator release from mast cells and provokes dyspnoea [130,131].

### 4.3. Der p 1 and Pannexon Gating

Under physiological conditions the concentration of ATP in extracellular fluid is low, but when elevated its presence as an “alarmin” signifies cellular distress and immunological activation. While various pathways could enable ATP release under stress (e.g., ATP binding cassette proteins, membrane damage), the dominant route activated by Der p 1 in airway epithelial cells appears to be the myosin motor gating of pannexons [78,103]. Pannexons are channels formed by hexameric assemblies of glycoproteins known as pannexins. In excitable tissues pannexons are voltage-gated, but this is an unlikely control mechanism in non-excitable cells in which membrane potential is unlikely to depolarise to levels to increase their open probability. Thus, other gating mechanisms, such as the biochemical activation of myosin motors, are required in epithelial cells. Unlike gap junctions (connexons), which are formed from connexin proteins, pannexon channels are unopposed, that is they do not form a contact with corresponding structures on neighbouring cells and cannot, therefore, participate in transcellular communication networks in the same way. Nevertheless, like gap junctions, pannexons are involved in the co-ordinated propagation of calcium waves through the airway epithelium following mechanical stimulation of a single cell, albeit through external cell–cell signalling rather than intracellular communication [132]. This transcellular signalling underlies several processes, notably the calcium-dependent co-ordination of ciliary beating, but pannexons are likely to be involved in the propagation of signals in addition to those which are primarily reliant on changes in intracellular [Ca^2+^].

The pannexin protein family comprises three members which associate most favourably in homomeric assemblies to form pannexons. Although heteromeric association can occur in model expression systems, scepticism surrounds the physiological occurrence of pannexons constituted in this way. Regardless of their constitution, gene silencing of pannexin-1 effectively inhibits the response of airway epithelial cells to Der p 1 [78]. Pannexons are permeant to divalent cations and serve as conduits for the diffusive movement of molecules <1 kDa. Thus, when pannexons open, cells release ATP to activate its receptors in an autocrine or paracrine fashion which enables continuity of the downstream signalling response [7,78]. In addition, ATP is thought to act either directly or indirectly on the pannexons themselves to provide feedback control of its own release. The permeability characteristics of pannexons suggest that ATP is unlikely to be the only mediator released from cells through this mechanism, and indeed the release of UTP is known to occur through pannexons in some cells [133]. However, beyond ATP the small-molecule mediator release aspect of pannexons has probably received less attention than it deserves in the two decades which have elapsed since their identification in the human genome. 

Myosin light chain kinase (MYLK) is a component of the gating of pannexons in airway epithelial cells whose activity is facilitated by the thrombin-dependent activation of PAR1/4 by Der p 1. Consequently, agents which modulate the activity of MYLK (such as ML-7, β_2_ agonists, or inhibitors of Rho-associated coiled-coil kinase) can attenuate cellular responses to Der p 1 to some extent [78]. Under greater duress, pannexons formed from pannexin-1 can be rendered into a constitutively open state by caspase 3 or caspase 7. This occurs because the lumen formed by the pannexon hexamer is partially occluded by the C-terminus of individual pannexin monomers and caspase-dependent truncation of each C-terminus results, therefore, in an open, unregulated channel which ultimately leads to apoptosis of the cell [133,134,135]. While Der p 1 can, independently of TJ cleavage, induce changes in the actin cytoskeleton of airway epithelial cells and promote apoptosis, the ability of Der p 1 to open pannexons and to generate ROS occurs without an obligatory caspase-dependent commitment to cell death [72,78].

Der p 1-dependent activation of PARs is not unique as a mechanism of pannexon opening relevant to asthma. Infection of cells by RNA viruses is sensed through ligation of pattern recognition receptors linked to signalling pathways which activate nuclear factor κB (NFκB) and interferon regulatory factor-3 (IRF-3) to result in altered gene expression. One pathway transducing these events is dependent upon the activation of TLR3 by endocytosed viral RNA, while another is operated by the retinoic acid inducible gene-I (RIG)-like receptors (RLRs) such as RIG-I and melanoma differentiation associated protein-5 (MDA-5). Both the TLR3 and RLR pathways are responsive to common respiratory viruses undergoing replication because these receptors sense double-stranded RNA. Ligation of TLR3, RIG-I, MDA-5 or the single-stranded RNA biosensor TLR7, all initiate ROS/RNS generation by airway epithelial cells and this too is dependent on the opening of pannexons [103]. In fact, evidence indicates that Der p 1 stimulation and pathways activated by viral RNA share the same downstream signalling pathways leading to ROS/RNS production and the generation of reactive signalling molecules which regulate gene expression [78]. Although the exact point of signalling convergence between Der p 1 stimulation and RLR activation is unknown, it appears to lie proximal to the events which regulate the gating state of pannexons and is independent of Janus kinase (JAK) signalling activated by viral RNA biosensor ligation [103]. It is noteworthy that PAR1 and TLR3 are both up-regulated by respiratory viruses associated with asthma exacerbations and that TLR3 stimulation per se induces RIG-like receptor expression in airway epithelium [113,136]. The cellular studies described above provide mechanistic insight into the ability of low doses of double-stranded RNA to induce airway inflammation in mice in a TLR3, IL-4 and STAT-6-dependent manner [137].

### 4.4. Der p 1 and ADAM 10

The activation of ADAM 10 in the downstream signalling cascade activated by Der p 1 adds a further dimension to the pleiotropic role of this metalloprotease in allergic diseases [79]. ADAM 10 was first associated with allergy through the discovery of its role as an ectodomain “sheddase” of the low affinity IgE receptor, CD23, on B-lymphocytes. CD23 is a C-type lectin whose soluble form enhances the presentation of antigen to allergen-specific B- lymphocytes, thus promoting the formation of allergen-specific IgE and an upregulated expression of IgE F_c_εRI [138]. Interestingly, this type of ectodomain shedding activity in CD23 has also been ascribed directly to Der p 1 [18]. The significance of ADAM 10 in ectodomain shedding from cells involved in responses to allergens is not restricted to CD23, however. Other targets of ADAM 10 include CCL20 (which recruits DCs and T_H_17 cells and promotes mucus hyperplasia), CCL2 (a chemoattractant for monocytes and dendritic cells), CCL5 (a chemokine for eosinophils), CXCL8 (a neutrophil chemokine), CXCL-16 (a T lymphocyte chemokine), IL-6R (cell differentiation and immunity), amphiregulin (EGFR ligand), tumour necrosis factor (acute phase cytokine), and heparin-binding EGF-like growth factor (involved in re-epithelialisation and smooth muscle mitogenesis) [139,140,141,142]. Additionally, ADAM 10 is a requirement for stem cell factor (SCF)-mediated recruitment of mast cells, suggesting it has a regulatory role in both populating mast cells with IgE and directing their spatial positioning within tissues [143]. Collectively, these effects encourage the view that ADAM 10 occupies a strategic role in allergy development at the interface between innate and adaptive immunity, and this concept is endorsed by clinical disease and in experimental models. Illustratively, ADAM 10 is over-expressed on B lymphocytes of people with asthma and, in experimental simulations of asthma its overexpression leads to the development of a disease phenotype (viz: mucus cell hyperplasia, airway constriction, inflammatory cell infiltration, and IgE production) [144,145].

As a putative signalling link in the Der p 1-to-ROS production cycle, ADAM 10 has several interesting features. First, it appears to be activated by ligation of either P_2_X_7_ receptors (P_2_X_7_R) or P_2_Y_2_R. Why there is a need for ATP to act through both a ligand-gated ion channel and a metabotropic receptor to achieve this is unclear and indicates that there may be situations where pyrimidine nucleotides (e.g., UTP) could be acting in preference to or in unison with ATP. In either case, it is reasonable to propose that receptor activation provides a necessary calcium signal for ADAM 10 ectodomain shedding activity, by opening the P_2_X_7_R channel which is permeable to mono- and divalent cations, and by activating protein kinase C and releasing Ca^2+^ from intracellular stores after ligation of P_2_Y_2_R. Second, the action of ADAM 10 in generating ROS in airway epithelial cells depends on prothrombin, prompting the question of whether this is a direct or indirect effect of ADAM 10 [79]. ADAM 10 is a member of the M12B metalloprotease sub-family which also includes the snake venom protease, ecarin, a known prothrombinase. The ability of ecarin to activate thrombin was exploited diagnostically in the ecarin clotting time test, an assay for anticoagulant status during treatment with hirudin. This establishes a precedent, but does not of course prove, that ADAM 10 could act as a prothrombinase like ecarin in epithelial cells activated by Der p 1. However, it should be noted parenthetically that ecarin certainly mimics ADAM 10 induced ROS production in airway epithelial cells, encouraging the parallel [79].

### 4.5. Der p 1 and Toll-Like Receptor 4

Epithelial cells express a wide range of TLRs consistent with the importance of mucosal surfaces in innate immunity. However, to avoid their triggering by minor threats to the host, the level of expression of TLRs is typically restricted at quiescence and/or their cellular distribution constrained by a basolateral polarisation [146]. Thus, cellular responses which produce material changes in this situation are early events in the detection of a perceived threat. One member of the TLR family, TLR4, has attracted particular attention in the context of allergic disease, both through the “hygiene hypothesis” in epidemiology and from mechanistic studies of immunology. The latter studies have demonstrated the importance of TLR4/MyD88 signalling and the priming action of IL-4, GM-CSF, and TNFα on CD4^+^ T cells in the development of allergy [4,29,147,148,149,150,151,152,153]. In mice, TLR4 expression on airway epithelial cells is an indispensable element in the development of sensitisation to HDM allergens as demonstrated by experiments using mice with conditionally mutant Tlr4 expression, or in irradiated bone marrow chimeric wild type and Tlr4^-/-^ mice [154,155,156,157]. This dependency arises because of the connection between TLR4, the activation of cells by IL-1α, and the release of IL-33 and GM-CSF [154,156]. Deficiency in TLR4 or neutralisation of this trio of cytokines inhibits allergy development [156].

**Figure 5:**
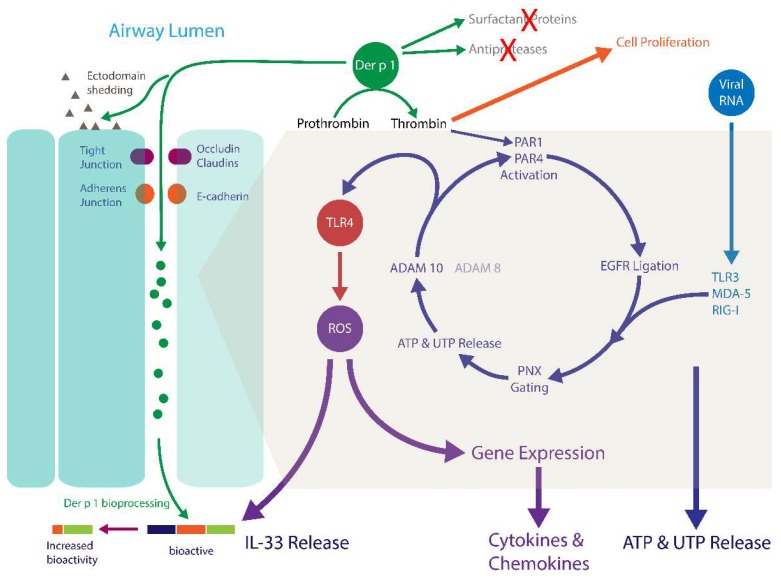
Schematic representation of a range of innate events triggered in airway epithelial cells by group 1 HDM allergens such as Der p 1. Proteolytic cleavage of tight junctions and adherens junctions facilitates transepithelial allergen delivery. Prothrombinase activity of the allergen leads to the canonical cleavage of PAR1 and PAR4, initiating a signalling cycle when enables the activation of TLR4 with the subsequent generation of ROS and the upregulation of inflammatory gene expression. Inactivation of airway defences (antiproteases and surfactant proteins) augment the effects outlined above. The release of the alarmin IL-33 from epithelial cells is facilitated by ROS and ATP and can be converted to a super-active form following cleavage by Der p 1.

Demonstrated using potent compounds designed specifically for the inhibition of Der p 1, and by the work of others using non-proprietary inhibitors, the protease activity of Der p 1 is crucial in establishing the commitment to IgE production when animals are sensitized to a mixture of HDM allergens [7,9,40]. This raises the question: what links the protease activity of Der p 1 with TLR4 activation? Recent studies in our laboratory have led to the proposal of a scheme which provides some explanation of this linkage between group I HDM allergens and TLR4. The key event is, once again, the unexpected prothrombinase activity of Der p 1 [7,78] (Figure 5).

The response of epithelial cells to Der p 1 or mixed HDM allergens is inhibited by gene silencing of TLR4, the prevention of TLR4 interaction with Toll-interleukin 1 receptor domain-containing adapter protein (TIRAP) and TRIF-related adapter molecule (TRAM), or the inhibition of Der p 1 protease activity [78]. Furthermore, the TLR4-dependent activation of ROS production is dependent on the opening of pannexons, ATP release, and prothrombin cleavage, consistent with the activation of TLR4 being downstream from the initial engagement of Der p 1 with epithelial cells [78]. In addition to recognising exogenous ligands, TLR4 is activated by a range of endogenous ligands among which are fibrinogen and cleavage products derived therefrom [158,159,160,161,162]. Because, airway epithelial cells are one of few tissues outside the liver where all three component chains (α, β, γ) of fibrinogen are expressed [163] and the effects of Der p 1 are coupled to TLR4 activation in an ADAM 10 and prothrombin-dependent manner, this suggested that fibrinogen and/or its cleavage products may be part of the ensemble which result in TLR4 ligation and ROS production. This interpretation is supported by significant reduction in the ability of Der p 1 to activate the production of ROS in epithelial cells in which the genes encoding the component chains of fibrinogen have been silenced individually [7]. Because pairs of individual fibrinogen chains (i.e., α,β; α,γ; β,γ) are unable to sustain the response when the third component is silenced [7], the functional requirement for this response is therefore a fully intact fibrinogen molecule comprised of α, β and γ chains. Interestingly, fibrinogen and its cleavage products have recently been identified as an innate activator of mast cells [158], providing further evidence for the concept that epithelial interactions with a variety of cells through TLR-dependent mechanisms are crucial to allergy initiation and maintenance.

Der p 2 has homology with the MD-2 co-receptor of TLR4 and can substitute the functions of the latter, suggesting that it might stimulate ROS production in a manner like LPS in phagocytes. However, Der p 2 is unable to activate significant ROS production in human airway epithelial cells with intact TJs [103]. The relative insensitivity of these cells to LPS, despite the presence of TLR4 in the cells, is consistent with a predominantly basolateral distribution of the receptor beneath the protection of TJs and it is this distribution of TLR4 which may explain an apparent paradox surrounding its prothrombinase-dependent activation. Given that TLR4 activation in airway epithelial cells involves thrombin activity, it could be anticipated that the prothrombinase activity of Der p 1 might activate TLR4 without any requirement for pannexon gating. However, this does not appear to be the case because the pannexon-dependent and thrombin-dependent nature of TLR4 activation implies a more distal connection between the allergen and TLR4. A possible explanation of this conundrum could lie in the cellular compartmentalisation of thrombin activation and its consequences. Until the cleavage of TJs allows otherwise through derogation of their “fence” function, it is probable that Der p 1 is initially restricted to exerting its prothrombinase activity on the apical surface of the cell where it is physically separated from the majority of TLR4 by the “fence” function of TJs. However, the downstream activation of thrombin which arises from the action of ATP and ADAM 10 may be compartmentalised, enabling prothrombin cleavage on the basolateral aspect of the cell, thereby generating thrombin in proximity to TLR4 and its activatable endogenous ligands. Alternatively, stimulation of PARs by thrombin generation might be comparatively transient, whereas ligation of TLR4 by the agency of products formed from endogenous prothrombinase activity is more sustained [78]. Further work will be necessary to investigate these, and other, possibilities.

**Figure 6:**
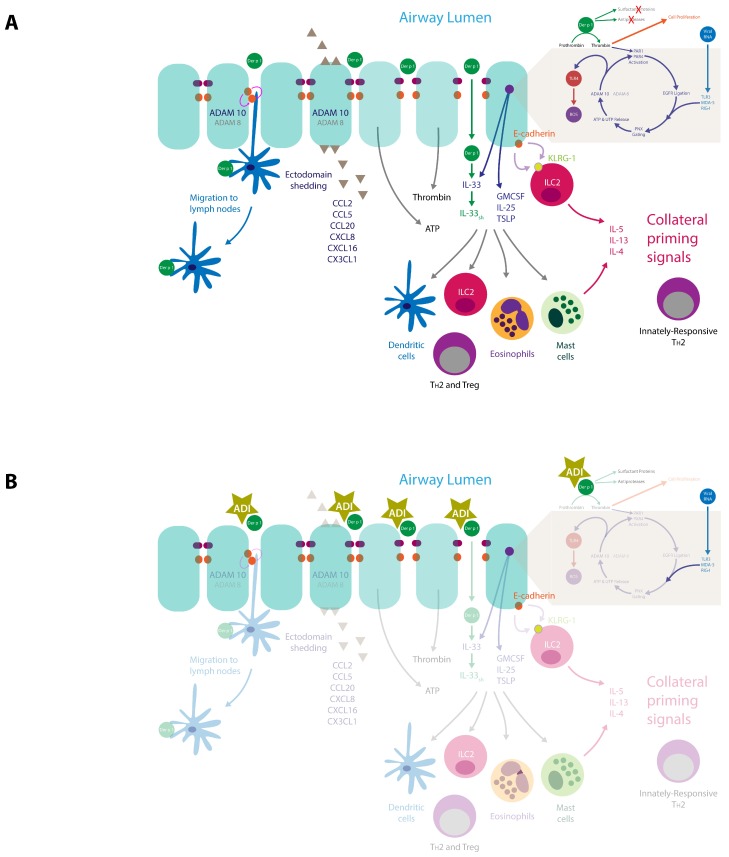
(**A**) Schematic representation of the consequences of the activation of innate signalling mechanisms in airway epithelial cells by group 1 HDM allergens. Please refer to text for full description of how the protease-initiated release of epithelial mediators coordinates a response of effector cells implicated in the initiation and progression of allergy. (**B**) Effects of allergen delivery inhibitor (ADI) compounds on the events depicted in (**A**) with inhibited pathways dimmed out.

Linkage of TLR4 activation to the protease activity group 1 HDM allergens is particularly interesting in respect of allergic polysensitisation. In mice, activation of sensitisation via TLR4 ligation results in IL-4 release by CD4^+^ T cells and IL-4Rα-dependent signalling. A corollary is that subsequent sensitisation to other allergens becomes independent of the requirement for TLR4 activation, at least while an inflammatory environment induced by the primary stimulus persists. This phenomenon has been dubbed “collateral priming” and signalling via the IL-4Rα sub-unit is a crucial component of the mechanism responsible [4]. Part of the significance of IL-4 is likely to be its capacity to inhibit the expression of indoleamine 2,3-dioxygenase, thus breaking immune tolerance [164]. Although the original body of work which defined this mechanism did not use HDM as the allergen source, there is strong similarity to the characteristics of HDM sensitisation. Indeed, it would be reasonable to infer that, as bioinitiators, the bioactivity profile of group 1 HDM allergens confers upon a single allergen molecule all the requisite properties (viz: TLR4 ligation, cytokine and chemokine production etc) required to encourage the development of sensitisation to unrelated allergens lacking these facilities, either from within the HDM allergen repertoire, or from entirely unrelated sources (Figure 6A).

The recent finding that group 1 HDM allergens execute a bioactivating cleavage of IL-33 suggests a mechanism which links these allergens to the activation of TLR4 in the airway epithelium and the liberation of a cytokine which is critical in the regulation of interactions between epithelial cells and ILC2s or innately-responding T_H_2 cells [5,6] (Figure 5). The capacity of the ILC2s and innately-activated T_H_2 cells to release IL-13 (which is ligated by the α-subunit of IL-4R, known to be important in collateral priming) [4], suggests how these processes may be associated, and it is noteworthy that ILC2s expanded by IL-33 enjoy a degree of longevity, a capacity for re-priming and exhibit a gene expression profile indicative of innate memory [5]. Given the epigenetic plasticity of innate responses of other cells to microbial ligands (“innate training”) it would be interesting to know if the altered properties of allergen-experienced ILC2s or innately activated T_H_2 cells extend to the acquisition of new functions too, although currently available data suggest that this might not be the case, at least for ILC2s [5]. The events described above may be the mechanisms which underlie the ability of group 1 HDM allergens to foster sensitisation to effete bystanders such as ovalbumin [102]. Present understanding of the possible roles of the overlapping innate priming effects of ILC2 cells and T_H_2 cells suggest that either perinatally, or in naïve mice lacking any allergenic exposure, it is the IL-33-driven ILC2 population which may facilitate the HDM allergen-dependent priming of responses to otherwise effete allergens, whereas in mice with sensitisation already developed it may be the IL-33-dependent, innate responses of T_H_2 cells which dominate [6,165,166]. Whatever the balance between these, and any other contributory events, their ultimate reliance on the protease activity of HDM allergen is suggested by the efficacy with which protease inhibitors prevent this polysensitisation (Figure 6A,B) [9].

## 5. Concluding Remarks

The past 25 years have seen a radical transformation of our understanding of allergy, its causes and underlying mechanisms. We have moved from a concept dominated by mast cells and IgE to one where the underlying engine of disease consists of a sophisticated network of cellular and molecular interactions which begin with epithelial cells and their neighbours. Such cellular interactions are facilitated by environmental allergen exposure and may be modified by a variety of genetic predisposing factors (barrier integrity, key mediator polymorphisms, abnormalities in checkpoint and countermeasure systems). These advances have paralleled the wider renaissance of innate immunity and the development of concepts such as innate “training” which have obvious relevance to diseases with chronic mechanisms. In terms of defining the molecular basis of allergenicity, an understanding of the intrinsic bioactivities of allergens has been shown to have deep relevance to identifying the innate immune pathways which they activate in humans to cause disease. This “functionalist” view of allergenicity provides understanding of well-known allergen phenomena, such as the concept of cross-reactive allergens [3]. More significantly, it has begun to provide unique insights into serodominance and the concept of initiator allergens. A corollary of the functional molecular hegemony of allergens and elucidation of the innate mechanisms they operate is the identification of novel molecular targets which lie upstream from the events against which existing small molecule therapies or emerging biologics are directed. A challenge in making novel interventions through regulation of these checkpoints will be the identification of those which match strong disease relevance with an acceptable safety profile. However, interventions directed towards the innate effector pathways themselves are just one option. As recently described [9], a small-molecule intervention against a carefully selected allergen target whose merits we have reviewed here and elsewhere [7] provides a range of benefits suggestive of an interestingly different therapeutic profile relevant to clinical needs.

While our purpose in this article has been to provide an overview focussed on group 1 HDM allergens, it is evident that several of the protease-dependent mechanisms described for these HDM allergens apply to protease allergens from entirely different sources. The centrality of these mechanisms to allergic disease suggests that proteolytic activity, which is confined to a discrete part of the allergome, is important in molecular hegemony. Consequently, they have attracted interest as innovative therapeutic targets and their inhibition has been shown to be associated with unexpected effects [7,9,10].

This returns us to an important practical point which is often overlooked in the thirst for new knowledge. Allergic mechanisms have a significant reliance on bioactive components of complex mixtures of unknown and variable composition acting in synergy to produce their effects and this justifies the use of mixed allergens in many biological experiments. However, commercial allergen extracts can vary significantly in their composition which affects their bioactivity profiles, and these may be modified further by the inclusion of additives to promote stability of products destined for clinical use. These factors introduce many potential traps for the unwary, as demonstrated by literature precedent, but these technical problems are addressable with appropriate awareness. Even with “purified” allergens we have seen biological mis-steps arising from the use of some reagents. With the advent of better tools there will be improved prospects for meaningful progress, and—at least in the case of the group 1 HDM allergens—the structure-based design of potent inhibitors provides the first truly selective tools with which to further explore the biology of these globally significant allergens [7,10,11].

## Figures and Tables

**Table 1:** Similarity matrix showing percentage identity between the mature protease forms of the house dust mite (HDM) group 1 allergens from *Dermatophagoides* and *Euroglyphus* species and cathepsin (cat) family members.

	Der p 1	Der f 1	Eur m 1	cat B	cat K	cat L2	cat S	cat Z
Der p 1	100	82	85	25	34	31	31	25
Der f 1	82	100	85	26	34	33	30	27
Eur m 1	85	85	100	25	34	32	30	25
cat B	25	26	25	100	28	30	33	24
cat K	34	34	34	28	100	59	58	28
cat L2	31	33	32	30	59	100	58	30
cat S	31	30	30	33	58	58	100	31
cat Z	25	27	25	24	28	30	31	100

## Abbreviations

ADAM	a disintegrin and metalloprotease
ADI	allergen delivery inhibitor
CCL	C-C chemokine ligand
CXCL	C-X-C chemokine ligand
DC	dendritic cell
Der f	*Dermatophagoides farinae*
Der p	*Dermatophagoides pteronyssinus*
EGF	epidermal growth factor
EGFR	epidermal growth factor receptor
GM-CSF	granulocyte macrophage-colony stimulating factor
GPCR	G-protein coupled receptor
GUK	guanylate kinase
HDM	house dust mite
IL	interleukin
ILC2	type 2 innate lymphoid cell
IRF-3	interferon regulatory factor-3
JAK	Janus kinase
JAM	junction-associated molecule
KLRG-1	killer cell lectin-like receptor group 1
LPS	lipopolysaccharide
MAPK	mitogen activated protein kinase
MD-2	myeloid differentiation protein-2
MDA-5	melanoma differentiation associated protein-5
MEKK1	mitogen activated protein kinase kinase kinase I
MYLK	myosin light chain kinase
NFκB	nuclear factor κB
Nrf2	nuclear factor (erythroid-derived 2)-like 2
PAR	protease-activated receptor
PI 3-K	phosphoinositide 3-kinase
PNX	pannexon
RIG-I	retinoic acid inducible gene-I
RLR	retinoic acid inducible gene-I (RIG)-like receptor
RNS	reactive nitrogen species
ROS	reactive oxidant species
RTK	receptor tyrosine kinase
SCF	stem cell factor
STAT	signal transducer and activation of transcription
TAMP	TJ-associated MARVEL domain protein
TER	transepithelial electrical resistance
Th2	type 2 T-helper cell
TIRAP	toll-interleukin 1 receptor domain-containing adapter protein
TJ	tight junction
TLR	toll-like receptor
TNFα	tumour necrosis factor α
TRAM	TRIF-related adapter molecule
TRIF	toll-interleukin 1 receptor domain-containing adapter-inducing interferon-β
Treg	regulatory T cell
TSLP	thymic stromal lymphopoietin
